# Hallucinations in borderline personality disorder: Prevalence, characteristics and associations with comorbid symptoms and disorders

**DOI:** 10.1038/s41598-017-13108-6

**Published:** 2017-10-24

**Authors:** Maria B. A. Niemantsverdriet, Christina W. Slotema, Jan Dirk Blom, Ingmar H. Franken, Hans W. Hoek, Iris E. C. Sommer, Mark van der Gaag

**Affiliations:** 1 Parnassia Psychiatric Institute, The Hague, The Netherlands; 2 Institute of Psychology, Erasmus University Rotterdam, Rotterdam, The Netherlands; 3 Rudolf Magnus Institute of Neuroscience, University Medical Center Utrecht, Utrecht, The Netherlands; 4 Department of Clinical Psychology and Amsterdam Public Health Research Institute, VU University, Amsterdam, The Netherlands

## Abstract

To establish the point prevalence of hallucinations in borderline personality disorder (BPD), telephone interviews were conducted with 324 outpatients diagnosed with BPD. Then a subgroup (n = 98) was interviewed in person to investigate the co-occurrence of these phenomena with other psychotic symptoms, comorbid psychiatric disorders, prior childhood adversities, and adult life stressors. For hallucinations in general a point prevalence of 43% was found, with rates for hallucinations in separate sensory modalities ranging from 8–21%. Auditory verbal hallucinations consisted mostly of verbal abuse and were generally experienced as distressing. A significant association was found between the severity of hallucinations on the one hand, and delusions and unusual thought content on the other; this association was absent for negative symptoms and disorganization. The presence of hallucinations also correlated with the number of comorbid psychiatric disorders, and with posttraumatic stress disorder (PTSD) specifically. Childhood emotional abuse and adult life stressors were also associated with hallucinations. The latter three associations suggest that patients with BPD might have an etiological mechanism in common with other patient/nonpatient groups who experience hallucinations. Based on these findings, we advise to treat PTSD *and* hallucinations when found to be present in patients with BPD.

## Introduction

In clinical practice, hallucinations experienced by patients with borderline personality disorder (BPD) are often designated as ‘pseudohallucinations’ to express the suspicion that they do not qualify as hallucinations proper^1^. One reason for this may be that the Diagnostic and Statistical Manual of Mental Disorders (DSM-5) states that, in the context of BPD, hallucinations occur “*only in some individuals during times of stress*”^2^. As a consequence, clinicians may be reluctant to label all positive disorders of perception in this group as hallucinations. However, studies during the last decade indicate that hallucinations proper are far from rare in patients with BPD, with prevalence rates ranging from 26–54%^3,4^. Moreover, these hallucinations are not restricted to a single sensory modality: 21–59% of them are auditory, 30–33% visual, 10–30% olfactory, and 13% tactile in nature^1,3,5–7^. In addition, they are often experienced as equally or more severe than those in patients diagnosed with a schizophrenia spectrum disorder^6,8^ and tend to be present for long periods of time, with a mean history for auditory verbal hallucinations (AVH) of 18 years^8,9^.

The number of studies on hallucinations in BPD is small, as is the sample size of most of those studies. Moreover, a limitation of the prevalence studies is the impossibility to generalize their results to the overall BPD population, as most of them focused on hospitalized patients^3,4,6,9^, solely AVH^1^, and lifetime prevalence rates^7^. As a consequence, our insights in hallucinations experienced by patients in the BPD group as a whole are still somewhat sketchy. Nevertheless, they are not as rudimentary as our insights into the occurrence of other psychotic symptoms in the context of BPD. Studies have found prevalence rates for delusions ranging from 10% to a full 100%^1,4,6,7^, whereas negative symptoms and disorganization have hardly been studied in this group. The few authors who did study them, found them to be far less prevalent in the context of BPD than in schizophrenia spectrum disorders^4,9^.

Regarding etiology, it has been suggested that comorbid psychiatric disorders might well constitute the true cause of hallucinations in BPD^10^. However, the evidence in support of that view is not univocal. Albeit four studies in the field of BPD found hallucinations and other psychotic symptoms to be associated with comorbid affective and substance-use disorders^4,10–12^, the presence of these disorders failed to predict any subsequent psychotic symptoms in a study by Miller *et al*.^13^ Moreover, Benvenuti *et al*.^14^ were unable to establish a difference between psychotic symptoms experienced by patients with BPD with and without a co-morbid mood disorder on a lifetime basis. Also noteworthy is the strong association between childhood adversity and psychotic symptoms in both patient and nonpatient groups^15^. The latter association was studied in patients with BPD by Tschoeke, Steinert, Flammer & Uhlmann^9^, who found a positive association between childhood trauma and suspiciousness, active social avoidance, and various specific characteristics of AVH in a group of 23 hospitalized patients with BPD.

Regarding the DSM-5 criterion which states that hallucinations in BPD are only experienced during times of stress^2^, we found only one empirical study to support that view, i.e. Glaser, van Os, Thewissen & Myin-Germeys^16^ reported that hallucinatory reactivity in response to self-reported daily life stresses was indeed significantly stronger in this group than in healthy controls and patients with a cluster C personality disorder.

To increase our knowledge on hallucinations in BPD, we studied patients from a specialized outpatient clinic for personality disorders while focusing on the following research questions:

1. What is the point prevalence of hallucinations in BPD in five sensory modalities, and what are their phenomenological characteristics?
2. Are hallucinations in BPD associated with other positive and negative symptoms of psychosis?
3. Are hallucinations in BPD associated with any comorbid psychiatric disorders?
4. Are childhood adversities and adult life stressors predictors of hallucinations in BPD?

## Methods

### Participants

From May 2012 through March 2015, all patients receiving treatment at the Outpatient Department for Personality Disorders at Parnassia Psychiatric Institute, The Hague, were approached by telephone for participation in this study. Inclusion criteria were 1) age ≥ 18 years; 2) a diagnosis of BPD, as established per May 1, 2012 in accordance with the operational criteria issued by the DSM-IV-TR^17^; 3) no comorbid DSM-IV diagnosis of schizophrenia or schizoaffective disorder, both ruled out on the basis of two clinical interviews by a trained psychologist and psychiatrist; and 4) sufficient mastery of Dutch or English. Patients were first approached by telephone, and then requested to be interviewed in person and fill out various self-report questionnaires.

### Instruments and procedure

During the telephone interview, which was tailor-made for this specific purpose, data were collected on the presence, content, and frequency of hallucinations. Each sensory modality was addressed separately, i.e., by asking whether the patient ever heard, saw, tasted, smelled or felt something that other people did not perceive, or for which they had no explanation. They were also asked about the frequency of these hallucinations and about their phenomenological characteristics. During the face-to-face interviews that followed, we used the following semi-structured interviews. The AVH-related subscale of the Psychotic Symptom Rating Scale (PSYRATS)^18^ was used to assess the phenomenological characteristics and ensuing distress of AVH, and the Positive and Negative Syndrome Scale (PANSS) was used to measure the severity of psychotic symptoms^19^. In conformity with the van der Gaag Five-Factor Model^20^, the PANSS data were limited to positive, negative, and disorganized symptoms. Only items that loaded at least nine times out of ten on the same factor were used. To establish the number of comorbid psychiatric disorders, and to identify any disorders considered capable of mediating hallucinations (i.e., unipolar depression, bipolar disorder, alcohol and drug abuse, and PTSD)^2,17,21^, we used the MINI-International Neuropsychiatric Interview (MINI PLUS 2000)^22^. The presence of childhood adversities and life stressors was assessed with the aid of two self-report questionnaires, i.e., the Childhood Trauma Questionnaire Short-Form, (CTQ-SF)^23^ and the Life Stressor Checklist-Revised (LSC-R)^24^. The CTQ-SF is designed to retrospectively assess five subtypes of childhood maltreatment, i.e., emotional abuse, sexual abuse, physical abuse, emotional neglect, and physical neglect, whereas the LSC-R assesses the presence and number of life stressors, which may vary from serious financial problems to being robbed or physically attacked. Assessors who carried out the telephone and face-to-face interviews were psychologists and residents of psychology and psychiatry who had been trained in conducting these specific interviews, and who during the inclusion phase participated in monthly meetings to safeguard the interrater reliability.

The study was approved by the National Medical Ethical Committee (Stichting Medisch-Ethische Toetsingscommissie Instellingen Geestelijke Gezondheidszorg; METiGG). Written informed consent was obtained from all patients who participated in the face-to-face interviews and filled out the self-report questionnaires. The study was carried out in accordance with all relevant guidelines and regulations. The datasets generated and/or analyzed during the current study are available from the corresponding author on reasonable request.

### Statistics

Statistical analyses were performed using IBM SPSS Statistics for Windows, version 23.0 (Armonk, NY: IBM Corp). Participant characteristics were compared between telephone and face-to-face interviews through independent samples T test (age), Mann-Whitney U test (GAF) and Chi square test (sex). The point prevalence of hallucinations was calculated by dividing the number of patients who experienced hallucinations at least once per month by the total number of patients that participated in the telephone interviews. As four patients failed to complete the telephone interviews, the denominator for calculating the point prevalence of the separate sensory modalities was 323 for auditory, 322 for gustatory and tactile, 321 for olfactory, and 134 for multimodal hallucinations. Because of tied ranks in the ordinal variables, we chose Kendall’s tau for correlation analyses to investigate the association between the severity of hallucinations (PANSS item P3) and other positive, negative, and disorganized symptoms (other PANSS items), as well as the number of comorbid psychiatric disorders and the presence of childhood adversities and adult life stressors. The Benjamini-Hochberg correction, allowing for a false discovery rate of 5%, was used after conducting multiple comparisons in the Kendall’s tau analyses of hallucinations and other positive, negative, and disorganized symptoms. To exclude childhood adversities from the analysis of adult life stressors, life stressors that had occurred before the age of 18 years were omitted. The association between the presence of hallucinations and the number of comorbid psychiatric disorders was analyzed with the Mann-Whitney U test, and between the presence of hallucinations and specific comorbid psychiatric disorders with logistic regression with backward Wald selection. Comorbid psychiatric disorders were clustered into three groups, i.e., mood disorders (unipolar depression, bipolar I and II disorder), PTSD, and substance-use disorders (alcohol abuse and dependence, drug abuse and dependence, or both). A proportional odds model with backward selection was used to analyze the association between the severity of hallucinations and specific comorbid psychiatric disorders, and between hallucinations and the five subtypes of childhood adversities. The odds ratios and their 95% confidence intervals were generated by converting the differences in log odds into the odds ratios via the Output Management System (OMS) Control Panel. The presence of hallucinations was defined as a PANSS item P3 score of  ≥ 4, and absence as a score of < 4.

## Results

**Figure 1:**
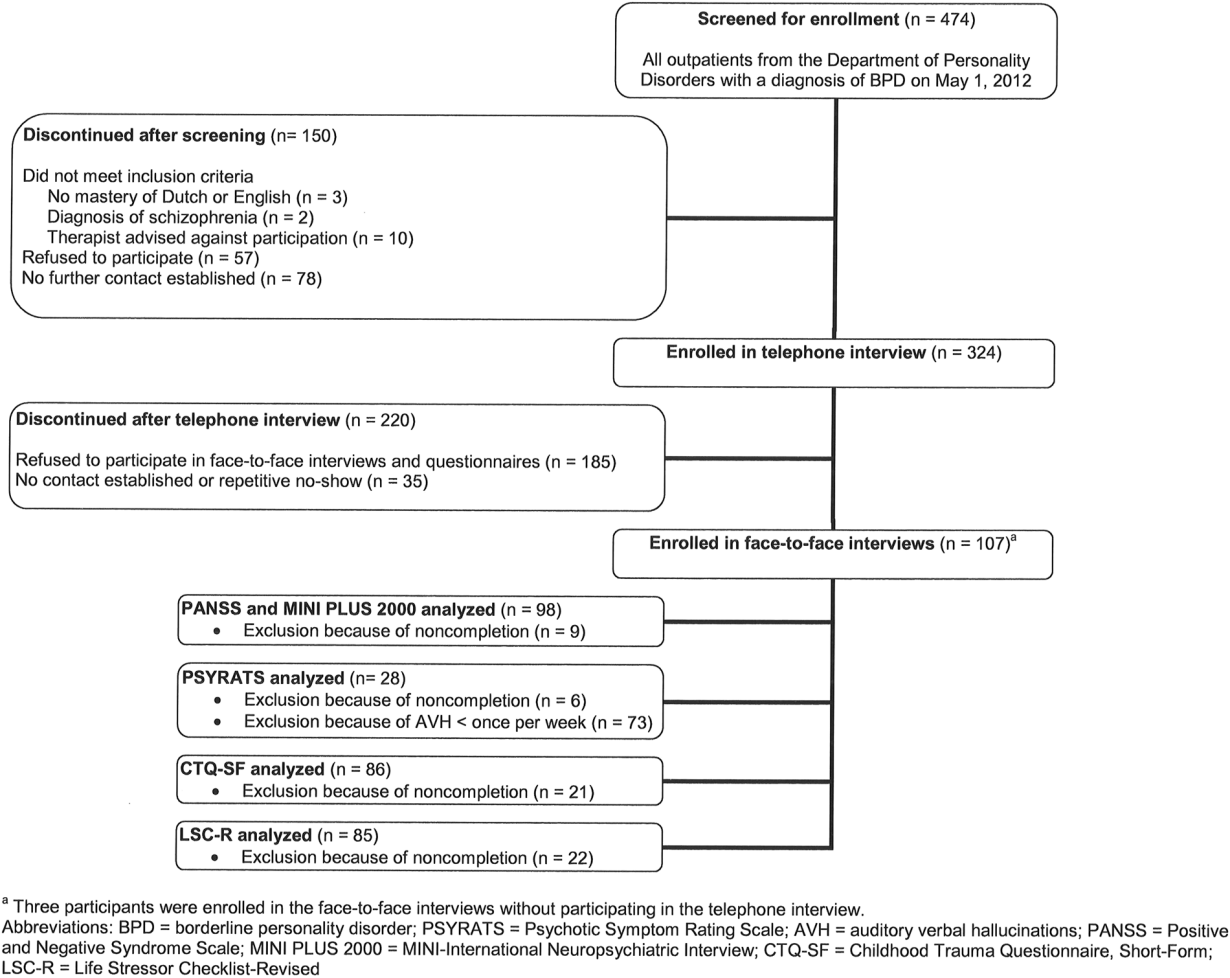
Flowchart participant disposition. The first panel shows all patients with a diagnosis of borderline personality disorder (BPD) from the Outpatient Department for Personality Disorders at Parnassia Psychiatric Institute who participated in the study. The next two panels show the patients that were excluded (discontinued) or included (enrolled) in the telephone interviews. Panels 4 and 5 display the patients who were excluded (discontinued) or included (enrolled) in the face-to-face interviews. Panels 6 through 10 show how many patients completed which questionnaires.

A total of 324 patients participated in the telephone interview. All of these patients were invited for subsequent face-to-face interviews, which were completed by 98 patients (Figure 1). Reasons for not completing were refusal to participate, failure on our side to establish further contact, and drop-out because of the duration of the interviews (which took 2–3 hours). Table 1 shows the participants’ demographic characteristics. There were no significant demographic and functioning differences between the patients who participated in the telephone interviews and the patients who participated in the face-to-face interviews.

**Table 1:** Participant characteristics.

Characteristic	Telephone interviews	Face-to-face interviews
N	324	107
Age, mean (SD)	37.4 (10.8)	37.3 (11.1)^a^
Female sex, n (%)	300 (92.6)	100 (93.5)^a^
GAF, median (range)	55 (40–80)	55 (40–80)^a^

Abbreviation: GAF = Global Assessment of Functioning.

^a^There were no significant differences between the two groups.

### Point prevalence of hallucinations and their phenomenological characteristics

Of the 324 patients who participated in the telephone interviews, 43% (n = 138) experienced hallucinations at least once per month, with a median frequency of at least once per week. Auditory hallucinations were reported by 87 patients (27%), including 69 (21%) who experienced AVH. In 50% of the cases (n = 67), the hallucinations were experienced in multiple sensory modalities. The PSYRATS interview was conducted among 28 of the patients who participated in the face-to-face interviews and experienced AVH at least once per week. Most of the AVH experienced by these patients had been present for long periods of time. Their content often involved verbal abuse directed at the patient, and they were mostly experienced as distressing in nature. Table 2 summarizes the prevalence rates of hallucinations as experienced in the five different sensory modalities that we studied. Table 3 presents the phenomenological characteristics of AVH and the distress caused by them.

**Table 2:** Point prevalence of hallucinations.

Hallucinations experienced at least once per month	n^a^	%	Example
Auditory (AVH)	87 (69)	27 (21)	Voice that gives assignments
Visual	37	11	Person, wraith or shadow
Gustatory	26	8	Taste related to past events
Olfactory	54	17	Smell of gas
Tactile	48	15	Sense of someone touching

^a^Four patients did not complete the telephone interview; the total sample consisted of 323 patients for auditory hallucinations, 322 for gustatory and tactile and 321 for olfactory hallucinations. Patients who experienced multimodal hallucinations were included in the calculation of the point prevalence of every sensory modality they experienced.

Abbreviation: AVH = auditory verbal hallucinations.

**Table 3:** Phenomenological characteristics of hallucinations (PSYRATS).

Characteristic of AVH	Item on PSYRATS	Score^a^	Description of score
Period, mean (SD), years	Additional question	15.3 (12.2)	
Number of different voices, median (range)	Additional question	2 (1–20)	
Voices of known person, n (%)	Additional question	17 (61%)	
Frequency, median (range)	1	3 (1–4)	Voices occur at least once per hour
Duration, median (range)	2	2 (1–4)	Voices last for several minutes
Location, median (range)	3	2 (1–4)	Voice close to or inside head
Loudness, median (range)	4	2 (1–4)	Same loudness as own voice
Explanation of origin, median (range)	5	3 (1–4)	Conviction that voice originates from external cause is 50%
Emotional valence, median (range)	6	3 (1–4)	Majority of content is unpleasant
	7	3 (1–4)	Personal verbal abuse related to self-concept
Total distress, median (range)	8	3 (1–4)	Majority of voices are distressing
	9	2.5 (1–4)	Voices are moderately to very distressing
	10	2 (1–4)	Moderate amount of disruption of life
Controllability, median (range)	11	4 (1–4)	No control over voices

^a^The sample consisted of 28 BPD patients who experienced AVH at least once per week.

Abbreviations: PSYRATS = Psychotic Symptom Rating Scale; AVH = auditory verbal hallucinations.

### Association with other positive symptoms, negative symptoms, and disorganization

Table 4 presents the median scores of the PANSS items, as collected during the face-to-face interviews, as well as the results of the correlation analyses. After correction for multiple comparisons, the only significant correlations were those between severity of hallucinations and delusions (tau-b = 0.371, p < 0.001), and between severity of hallucinations and unusual thought content (tau-b = 0.330, p < 0.001). Item scores for unusual thought content, however, did not reach the cut-off point of 4 (presence of symptoms), which makes this correlation less likely to be clinically relevant. After correction, correlations with negative symptoms and disorganization were both non-significant.

**Table 4:** Association between hallucinations and other positive symptoms of psychosis, negative symptoms, and disorganization.

		Item score, median (%)^a^		tau_b	p-value
		Hall −^b^ (n = 71)	Hall+^c^ (n = 27)		
PANSS-POSITIVE	P1 Delusions	1 (8.5)	3 (44.4)	0.371	<0.001^d^
	P3 Hallucinatory behavior	—	—	—	—
	P5 Grandiosity	1 (1.4)	1 (0)	0.046	0.614
	P6 Suspiciousness/persecution	3 (21.1)	3 (37.0)	0.208	0.013
	G9 Unusual thought content	1 (0)	2 (0)	0.330	<0.001^d^
PANSS-NEGATIVE	N1 Blunted affect	1 (4.2)	1 (7.4)	0.141	0.113
	N2 Emotional withdrawal	1 (5.6)	1 (7.4)	0.097	0.267
	N3 Poor rapport	1 (1.4)	1 (3.7)	0.071	0.434
	N4 Passive/apathetic social withdrawal	2 (4.2)	2 (11.1)	0.228	0.008
	N6 Lack of spontaneity and flow of conversation	1 (2.8)	1 (3.7)	−0.031	0.737
	G7 Motor retardation	1 (0)	1 (3.7)	0.064	0.475
	G8 Uncooperativeness	1 (0)	1 (0)	0.022	0.809
	G16 Active social avoidance	2 (8.5)	3 (18.5)	0.206	0.015
PANSS-DISORGANIZATION	P2 Conceptual disorganization	1 (1.4)	1 (7.4)	0.206	0.022
	N5 Difficulty in abstract thinking	1 (4.2)	1 (0)	−0.027	0.757
	N7 Stereotyped thinking	1 (0)	1 (0)	0.153	0.092
	G5 Mannerisms and posturing	1 (2.8)	1 (0)	0.141	0.122
	G10 Disorientation	1 (0)	1 (3.7)	0.137	0.128
	G11 Poor attention	1 (4.2)	2 (7.4)	0.098	0.263
	G12 Lack of judgement and insight	1 (0)	1 (7.4)	0.095	0.287
	G13 Disturbance of volition	1 (1.4)	1 (0)	−0.083	0.352

^a^The sample consisted of 98 patients with BPD who completed the PANSS interview.

^b^Hallucinations absent, defined as PANSS item P3 score <4.

^c^Hallucinations present, defined as PANSS item P3 score ≥4.

^d^Statistically significant after Benjamini-Hochberg correction.

Abbreviation: PANSS = Positive and Negative Syndrome Scale.

### Association with comorbid psychiatric disorders

Patients with BPD who did experience hallucinations (median = 5) had received significantly more comorbid psychiatric diagnoses than those who did not (median = 2; U = 536.500; z = −3.379, p = 0.001). In addition, the severity of hallucinations in these patients was associated with the number of comorbid psychiatric disorders (tau-b = 0.374, p < 0.001). Sixty-three percent (n = 17) of patients *with* hallucinations met criteria for PTSD, against 28% (n = 20) of patients without hallucinations. The association between both the presence *and* severity of hallucinations and PTSD was significant (OR 5.051, CI 2.193–11.628, p < 0.001, OR 3.311, CI 1.237–8.863, p = 0.017), whereas the association between the presence and severity of hallucinations and mood and substance-use disorders was not.

### Association with childhood adversities and adult life stressors

Positive and significant correlations were found between the severity of hallucinations and preceding childhood adversities (tau-b = 0.200, p = 0.014). The odds ratio for higher scores on emotional abuse (as compared to lower scores) to experience more severe hallucinations was 1.237 (CI 1.116–1.371, p < 0.001), whereas the reverse was found to be true for emotional neglect (OR = 0.867, CI 0.785–0.958, p < 0.001). The subtypes sexual abuse, physical abuse, and physical neglect were not significantly associated with the severity of hallucinations. Finally, a greater number of current life stressors correlated positively with more severe types of hallucination (tau-b = 0.275, p = 0.001).

## Discussion

Our analysis of 324 outpatients diagnosed with BPD yields a point prevalence for hallucinations of 43%, with half of the hallucinations being multimodal in nature. In comparison with prevalence rates of hallucinations in the general population (3–6%)^25,26^ and in schizophrenia (75%)^27^, BPD thus seems to occupy some middle ground on this spectrum of nosology. However, regarding the phenomenological characteristics of their hallucinations, these patients seem to fall squarely in the severe end of the spectrum. In a review by Johns *et al*.^28^, the authors describe the difference between benign types of hallucination and pathological ones (neutral vs. negative content, high vs. low control, and low vs. high frequency). The hallucinations experienced by patients participating in the present study fall in the latter category. This is in line with an earlier study, reporting that the phenomenological characteristics of hallucinations in patients with BPD do not differ significantly from those in patients with schizophrenia^6,8,9^, and that the ensuing distress is often even higher^6^. In the present study, although hallucinations experienced by patients with BPD were often found to co-occur with delusional thinking, they did not tend to be accompanied by negative symptoms and disorganization. In clinical practice, this may help to distinguish between patients with BPD and those with schizophrenia^2,29^_._

As the *number* of comorbid psychiatric disorders was associated with the presence and severity of hallucinations, and PTSD was the only specific comorbid psychiatric disorder for which this association was established, we consider it unlikely that hallucinations in this patient group are caused by any particular comorbid psychiatric disorder, as has been suggested^4,10–12^, at least not in the majority of them. Even the presence of comorbid PTSD does not seem to provide a sufficient explanation for the presence of hallucinations in BPD, since 37% of the BPD patients who experienced hallucinations did not meet the diagnostic criteria for PTSD. If we are allowed to consider a greater number of comorbid disorders as an index of illness severity, then patients with BPD and hallucinations seem to belong to a subgroup with a more severe type of personality disorder. If this is correct, this would be in line with Glaser *et al*.^16^, who found a dose-response relation between the number of BPD symptoms and psychotic reactivity.

Another noteworthy finding from the present study is the correlation between prior childhood adversities and the present severity of hallucinations, especially when those adversities involve emotional abuse. Such a relation between childhood adversities and a future chance of developing psychotic symptoms has been reported^15^, but not specifically for patients with BPD. Our finding that the odds ratio for the severity of hallucinations is higher for emotional than for sexual and physical abuse is in line with others^30,31^, and underlines the importance of exploring this type of abuse in patients (whatever their diagnosis) who experience hallucinations. Interestingly, we found an inverse relationship between the severity of hallucinations and prior emotional neglect. Two studies on patients at ultra-high risk for psychosis found similar results, i.e., a positive association between prior emotional abuse and the chance of transition to psychosis in the first^32^ and the severity of positive psychotic symptoms in the second study^33^, plus a negative association between emotional neglect on the transition to psychosis^32^ and (albeit non-significant) the severity of positive psychotic symptoms^33^. Together, these findings suggest that a history of childhood emotional abuse increases the susceptibility for hallucinations (often with a content directly/indirectly related to the traumatic experience)^21^, whereas emotional neglect does not appear to have that effect. Although the underlying mechanisms remain to be elucidated, it is hypothesized that the susceptibility for psychosis might be lowered when children learn that (even without noteworthy emotional support) they are still able to acquit themselves^32^.

Regarding the role of stress in promoting hallucinations in BPD, our study highlights the need for a discussion that strikes more of a balance than the traditional ones. Glaser *et al*.^16^ found hallucinatory reactivity in patients with BPD in response to ‘daily hassles and minor stresses’, while our study indicates that the severity of hallucinations is associated with adult life stressors. However, it seems that this type of response to stress is not specific for BPD. Various studies report the pivotal role of psychosocial stresses on hallucinations and other psychotic symptoms in healthy individuals, in individuals with an at-risk mental state, and in patients diagnosed with a schizophrenia spectrum disorder^34,35^. We consider this an argument in favor of the ideas i) that hallucinations in the context of BPD do not differ significantly from those in other disorders, ii) that they may even share a common etiology, and iii) that investigation of these hallucinations may benefit from a transdiagnostic approach. A further argument for the transdiagnostic approach stems from our finding that in BPD, PTSD was the only comorbid psychiatric disorder associated with the presence *and* severity of hallucinations. In a comprehensive review, McCarthy *et al*.^21^ describe the various phenomenological similarities between AVH experienced in the context of schizophrenia and in PTSD, as well as a possible shared etiology involving traumatic experiences; the authors also propose a transdiagnostic approach to AVH. Given our finding that these phenomenological similarities also exist for AVH in patients with schizophrenia and with BPD^6,8,9^, and given the associations we found between hallucinations and childhood adversities and PTSD, we believe that the hypothesis of a common etiology for AVH in BPD, schizophrenia and PTSD warrants further study.

### Limitations

An initial limitation of the present study is that, in our outpatient group, we established the point prevalence of hallucinations based on telephone interviews. As some of the participants seemed reluctant to discuss their symptoms freely during telephone conversations, this may have led to an underestimation of the actual prevalence figure. In the final analysis, of the 11 patients who stated during telephone interviews that they had *never* experienced hallucinations, all later admitted during the ‘live’ PANSS interview that they had. Conversely, 12 patients who stated over the telephone that they *had* experienced hallucinations, failed to meet the criteria for these phenomena during the PANSS interview. However, as these numbers are very similar, the point prevalence we established may still have been reasonably accurate. On the other hand, in patients with BPD, these discrepancies may indicate the instability of hallucinations over time, especially since some patients had face-to-face interviews several months or even up to two years after their telephone interview. Consequently, we may have to conclude that the point prevalence of hallucinations in this patient group is *a priori* subject to some change.

A second limitation is our use of questionnaires (e.g. the PSYRATS and the PANSS) which are validated for patients diagnosed with schizophrenia, but not for use in those with BPD. To our knowledge, however, no questionnaires validated specifically for use in patients with BPD are currently available.

A third limitation is the strikingly high percentage of female participants in our study (93%), which might limit the generalization of our findings to male patients with BPD. This despite that BPD is far more common in women than in men, i.e. on average 25% of BPD patients are male^2,36^. That said, in the present study, the female/male ratio was an accurate representation of the population under care at our Outpatient Department for Personality Disorders where, in 2012, women made up 92% of the population.

A final limitation is that we did not focus on dissociative symptoms and disorders. Dissociation is common in patients with BPD^37^, and a comprehensive review and meta-analysis by Pilton, Varese, Berry & Bucci^38^ suggests a strong relationship with AVH, as well as with other types of hallucination. Dissociation might be an important mediating factor between childhood adversities and hallucinations in patients (whatever their diagnosis) and healthy individuals. Therefore, for future studies, our advice is to take these symptoms and disorders into account when investigating the mechanisms underlying hallucinations in patients with BPD.

### Further recommendations for research

Although the prevalence, phenomenological characteristics and comorbidity of hallucinations were adequately addressed with our cross-sectional approach, even more accurate data on causality, the role of childhood adversities, the role of life stressors, and the role of dissociation can be obtained by means of prospective studies. In doing so, the interactions and associations between BPD, PTSD and dissociation in patients who develop hallucinations are of particular importance.

Further research might also focus on delusions in patients with BPD, which constitute yet another group of underexposed symptoms. In addition, the inverse relationship between emotional neglect and hallucinations deserves further attention. Finally, studies on treatment for hallucinations in patients with BPD are needed, to examine whether these patients can benefit from the same interventions as used in patients with schizophrenia spectrum disorders, i.e., antipsychotics, cognitive-behavioral therapy, eye-movement desensitization and reprocessing, and transcranial magnetic stimulation^39,40^.
